# Nest‐building activity as a reproducible and long‐term stroke deficit test in a mouse model of stroke

**DOI:** 10.1002/brb3.993

**Published:** 2018-04-27

**Authors:** Dong Yuan, Chunli Liu, Jiang Wu, Bingren Hu

**Affiliations:** ^1^ Department of Anesthesiology and Neurology Shock Trauma and Anesthesiology Research Center University of Maryland School of Medicine Baltimore Maryland; ^2^ Department of Neurology The First Hospital of Jilin University Changchun China; ^3^ Veterans Affairs Maryland Health Center System Baltimore Maryland

**Keywords:** behavior test, cognition, functional test, histopathology, MCAO, mouse stroke, mouse stroke model, nest‐building activity, sensorimotor function deficit and recovery, well‐being

## Abstract

**Introduction:**

Neuroprotective therapeutics achieved from animal studies have not been able to translate into clinical stroke therapies. A major reason may be that the functional tests and outcomes between animal stroke studies and clinical trials are significantly different. Ultimately, functional recovery is most important for stroke patients, but it remains challenging to identify animal functional tests that reflect human stroke deficits. This study aimed to explore whether the nest‐building activity can be used as a functional test of mouse stroke deficit.

**Methods:**

Forty‐one C57B6 male mice were randomly assigned into a sham‐operated control group and 20‐, 40‐ and 60‐min middle cerebral artery occlusion (MCAO) groups. Mice were perfusion‐fixed at 21 days following sham surgery or MCAO. Infarct volumes were assessed under the light microscopy. The nest‐building activity was characterized and quantitatively evaluated.

**Results:**

The results show that only a small portion of striatum was damaged after 20‐min MCAO. The brain damage areas were expanded from striatum to the neocortex and hippocampus proportionally after 40‐min and 60‐min MCAO, respectively. Consistently, relative to that of the sham‐operated mice, the nest‐building activity was insignificantly altered after 20‐min MCAO, but dramatically and significantly reduced proportionally following 40‐min and 60‐min MCAO, respectively. The nest‐building deficit was a long‐lasting event and could be seen for as long as 14‐21 days of recovery, the longest endpoint of this study.

**Conclusions:**

The results suggest that the nest‐building activity may be a novel, objective, easy to use, highly sensitive, and long‐lasting test that may reflect the multifaceted sensorimotor and cognitive deficits after stroke in humans. Our findings may provide a novel multifaceted test for bridging the gap between animal stroke studies and clinical trials.

## 1 INTRODUCTION

Ischemic stroke is a major cause of death and disability worldwide. Significant efforts and resources have been devoted to experimental stroke research for understanding the underlying mechanisms and for developing clinical therapies. However, these efforts and resources have not been able to translate into effective clinical therapies (Fisher, 2011). A major reason may be the significant difference in the evaluation of the functional deficits between animal studies and stroke clinical trials. Many functional or behavioral tests in animal studies are not to assess natural animal behaviors; rather animals are forced to perform a task designed by investigators. Sensorimotor dysfunction is the most prominent clinical stroke deficit, but rodents are able to adapt and thus recover from sensorimotor deficits quickly after stroke (Rewell et al., 2017). As a result, some sensorimotor tests may be less sensitive, especially at the late time points, in rodent models of stroke (Balkaya, Kröber, Rex, & Endres, 2013; Balkaya et al., 2017). Therefore, there is unmet need of developing effective natural functional assessments for animal studies that reflect both short‐ and long‐term deficits of human stroke patients (Encarnacion et al., 2011).

Nest building is a natural behavior throughout the animal kingdom (Bachstetter et al., 2014). Primates such as extant strepsirrhines (lemurs and lorisoids) and hominid apes (gorillas, chimpanzees, and orangutans) build nests for both sleeping and also for raising families (Stewart, Pruetz, & Hansell, 2007; Stewart, 2011; van Casteren et al., 2012). Small rodents build nests for bed and shelter. Nest‐building behavior in animals may be similar to preparing bed and maintaining houses in humans (Lin, Chen, Kuang, Wang, & Tsien, 2007; Bachstetter et al., 2014). Normal nest‐building activity also indicates an animal's well‐being and, perhaps most importantly, is performed spontaneously in the home cage without enforcement by investigators (Jirkof, 2014).

The objective of this study was to investigate whether nest‐building activity can be used as a novel, quantitative, sensitive, and long‐term functional test in the mouse model of stroke. The results convincingly show that mouse natural nest‐building activity can be easily, objectively, and quantitatively analyzed with a pressed cotton nestlet in the home cage. Unlike some functional tests, mouse nest‐building activity is a spontaneous behavior without the need for human enforcement. It is a simple, cheap, and quantitative test and can be used to assess mouse long‐term sensorimotor function, cognitive function, and well‐being after stroke. Mouse nest‐building activity test is likely to become a sensitive method for measuring the degrees of stroke deficits and recovery.

## 2 MATERIALS AND METHODS

### 2.1 Ethics statement

The study was conducted in accordance with National Institutes of Health guidelines for the use of experimental animals. The animal protocols were approved by the Animal Care and Use Committee at the University of Maryland, Baltimore.

### 2.2 Animals

Male C57B6 mice, age about 10 ‐ 12 weeks, were purchased from Charles River Laboratories (Wilmington, MA). Mice were given food and water ad libitum and kept on a 12‐hr light/dark cycle in climate‐controlled housing. Animals were cared according to institutional guidelines in the animal resource facility at the University of Maryland Baltimore.

### 2.3 Middle cerebral artery occlusion (MCAO) model

Before surgery, mice were assigned to experimental groups. The conditions for exclusion, endpoint criteria, euthanasia, and sample size were defined also before surgery. Male C57B6 mice were subjected either to 0 (sham surgery, *n* = 11), 20 min (*n* = 6), 40 min (*n* = 14), or 60 min (*n* = 10) MCAO followed by 21 days of reperfusion according to the method of Zhang, Graham, and Chen (2009). Mice were anesthetized with isoflurane, 5% induction, and 1.5% maintenance. To ensure adequately ischemia and reperfusion, cerebral blood flow in the middle cerebral artery (MCA) territory was carefully and continuously monitored with laser Doppler flowmetry (LDF) (PowerLab, ADinstruments, Colorado Springs, CO) before, during, and after MCAO. An incision was made in the area between the ear and the eye to expose the temporal bone for inserting a needle laser Doppler probe. The Doppler probe was affixed to the skull of the MCA territory for the entire period of surgery under anesthesia. The mice were then placed in the supine position. An incision was made in the midline neck region, and the common carotid artery (CCA) and left external carotid artery (ECA) were isolated. Left ECA was then permanently ligated. A 12‐mm monofilament (6‐0, nylon suture, United States Surgical, Norwalk, CT, USA) was coated with silicone. The sizes of the final coating diameter of 0.22‐0.23 mm and coating tip length of 2.0 ± 0.2 mm were carefully and precisely selected. The silicone‐coated suture was inserted into the ECA and further advanced via the internal carotid artery (ICA) to the base of the MCA to block the blood flow to the MCA territory. MCA occlusion was confirmed during the entire MCAO period by the blood flow measurement via the LDF (PowerLab, ADinstruments, Colorado Springs, CO) as described above. After 0 (sham)‐, 20‐, 40‐, or 60‐min MCAO, the monofilament suture was removed. The reperfusion was confirmed by the corresponding return of the MCA blood flow to the about preocclusion level via the LDF. After 70 min (for the sham group), 50 min (for the 20 min MCAO group), 30 min (for the 40 min MCAO group), and 10 min (for the 60 min MCAO group) of reperfusion, isoflurane was discontinued, all wounds were sutured, and mouse was returned to the home cage. For the survival period, the mouse was kept in the cage with facilitated access to water and food. Sham‐operated mice received the same surgical procedure except the monofilament was inserted and immediately removed. During the surgery, mouse body and mouse head temperature was monitored and maintained at 37 ± 0.5°C with a warm water blanket and a heating lamp. The mice were allowed to survive for 21 days postsurgically.

### 2.4 Experimental groups

Prior to the surgical procedure, mice were randomly and blindly assigned to either sham (*n* = 11), or 20‐min (*n* = 6), 40‐min (*n* = 14), and 60‐min (*n* = 10) MCAO groups, followed by 21 days of reperfusion. These sample sizes were determined based on the power analysis of the data from our own studies.

### 2.5 Exclusion and inclusion

All mice were included except one mouse in the 40‐min sham surgery group because of the data belonged to the outlier based on the Grubbs’ test statistical analysis.

### 2.6 Nest‐building activity

All mice were randomly assigned to experimental groups before surgery. All analyses were performed blindly. Mice were singly housed with wood‐chip bedding with no environmental enrichment items and had ad libitum access to food and water throughout the experiments. Mouse performed nest‐building activity naturally and autonomously in the “home” cage. Normal mice only need less than 10 min to build a nest (Rock et al., 2014). The nestlet is a small piece of pressed cotton for a mouse to make a nest in his or her home cage (see below Figure 3 a, left). The nestlet used in this study was a 5 cm square of pressed cotton batting (Ancare, Bellmore, NY). The nestlet was manufactured from pulped virgin cotton fiber, sterilized during manufacture, and cleanly packed. Nestlets do not deteriorate during storage. Only 2.5 g nestlets were used. If heavier than 2.5 g, nestlets were trimmed to 2.5 g before use. One piece of nestlet was placed in the corner of each cage at 10 a.m., The next morning at 10 a.m., the amounts of torn and untorn nestlet materials in each cage were weighed. An untorn piece was defined as that heavier than 0.05 g. The percentage of torn vs. untorn nestlet materials was then calculated. If a nestlet was all torn, it was counted as 100%. If a nestlet was completely untorn, it was counted as 0%. The percentage of torn nestlet was counted as 0% after mice were dead.

### 2.7 Quantitative histological analysis of brain injury area and volume

Quantitative analysis of brain damage area was performed upon completing the nest‐building activity tests. After 3 days following MCAO, the amount of brain damage size becomes relatively stable (Rewell et al., 2017). The definition of the total damaged brain region includes both the core and penumbral area. The borderline between the normal area containing only normal neurons and damaged area containing any recognizable dead neurons was delineated. Briefly, mice were anesthetized and perfusion‐fixed via the ascending aorta with 100 ml ice‐cold phosphate‐buffered saline (PBS) and then 200 ml of 4% paraformaldehyde in PBS. Brains were postfixed for 3 days and sectioned consecutively at 50 μm thickness between bregma +1.54 mm and −2.31 with a Leica VT1000 S vibratome (Leica Biosystems, Nussloch, Germany). Up to 77 sections were collected and stored individually in the wells of a standard 96‐well polypropylene plate. The wells were half‐filled with an antifreeze solution. Brain sections were stored at −20°C until use.

Quantitative histopathological analysis of brain damage size was performed with a method described in our recent publications (Kristian & Hu, 2013; Luo et al., 2015). Every tenth 50‐μm‐thick section starting from bregma +1.35 mm and a total of 8 sections were selected. All eight brain sections were mounted onto a single microscopic glass slide and stained with hematoxylin and eosin (H&E). A total of 20 (for the smallest striatal section) ‐ 30 (for the largest hippocampal section) individual histological images from each brain section were captured under a 5x objective of the Nikon Eclipse 800 microscope equipped with a camera system. A montage of the entire brain section was then assembled with the StereoInvestigator program (Kristian & Hu, 2013; Luo et al., 2015; ). The total normal areas in both contralateral and ipsilateral hemisphere were separately quantified with the NIH ImageJ software. The total damage area in each section was calculated with a formula; total damaged area = contralateral normal area – ipsilateral normal area. The total damage volume was calculated based on eight brain sections at 0.5 mm apart ranging from 1.35 to −2.15 mm relative to bregma. For the total damage volume, we first calculated each damage volume between two adjacent sections using the average area multiplying the distance between the two adjacent sections. For example, the first damage volume = [(the damage area of the first section + the damage area of the second section)/2 × the distance between the sections (0.5 mm)]. The total damage volume was then calculated by adding up all the 7 damage volumes among eight sections.

### 2.8 Statistics and analysis

KaleidaGraph software (Synergy Software, PA, USA) was used for the statistical analysis. The data distribution of brain damage areas or volumes is likely nonparametric (Marchal et al., 1999). For the nest‐building test, most sham‐operated mice have 100% nestlet material torn, whereas MCAO mice having 0%‐100% torn. Therefore, the data distribution is also likely nonparametric. Therefore, Mann–Whitney Wilcoxon analysis was used for the statistical analysis of both brain damage size and nest‐building activity. Results were presented as means ± *SEM* for the indicated number of experiments (*n*). *p* value equal to/less than .05 was considered to be statistically significant.

## 3 RESULTS

### 3.1 Brain damage size after MCAO

**Figure 1:**
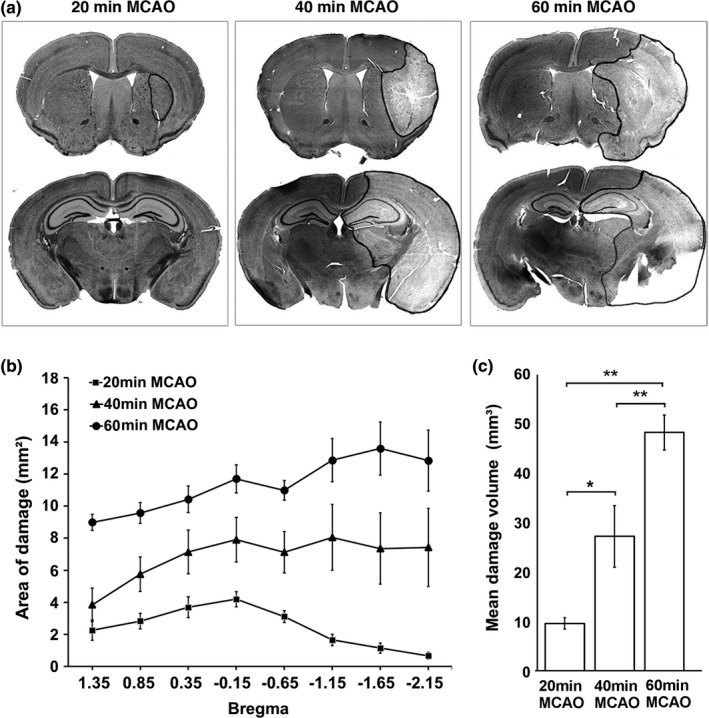
(a) Representative coronal sections of mouse brains at the striatal and hippocampal levels after 20‐, 40‐, and 60‐min middle cerebral artery occlusion (MCAO), respectively. The borderline between the area containing only normal neurons and damaged tissue containing any recognizable damage neurons was delineated with a solid black outline. (b) Quantitative analysis of brain damage areas at different brain section levels relative to bregma in 20 (*n* = 6), 40 (*n* = 8), and 60 (*n* = 5) min of MCAO groups. (c) Quantitative analysis of brain damage volumes in 20 (*n* = 6)‐, 40 (*n* = 8)‐, and 60 (*n* = 5)‐min MCAO groups. Data (b and c) are expressed as mean ± *SEM* for the indicated number of experiments (*n*). Mann–Whitney Wilcoxon analysis was used for the statistical analysis. *p <* *.05, **p < .01

To evaluate the extent of brain damage areas and volumes following MCAO, we stained brain sections with H&E. We defined the total brain damage area where contains any damaged neurons under the light microscopy (including both the core and penumbral regions). Figure 1a shows the striatal (−0.15 mm from bregma) and hippocampal (−2.15 mm from bregma) brain sections. As expected, in this mouse MCAO model, 20‐min MCAO damaged only a small portion of the striatum, and 40 min of MCAO led to not only striatal but also neocortical and hippocampal structural damage, while 60 min of MCAO resulted in more expanded striatal, neocortical, and hippocampal structural damage in the ipsilateral brain hemisphere (Figure 1a). Normal neurons were characterized by the large round neuronal nuclei stained in a light purple color with hematoxylin and cell bodies stained in a light pink color with eosin (Yuan, Liu, & Hu, 2018). The neuropil areas were stained uniformly also in the light pink color with eosin (Figure 1a). Dead neurons showed significantly shrunken and polygonally shaped and darkly stained or basophilic nuclei and with acidophilic or thick pink cytoplasm (Yuan et al., 2018). The borderline between the area containing only normal neurons and damaged tissue containing any recognizable damage neurons was delineated as shown in Figure 1a.

**Figure 2:**
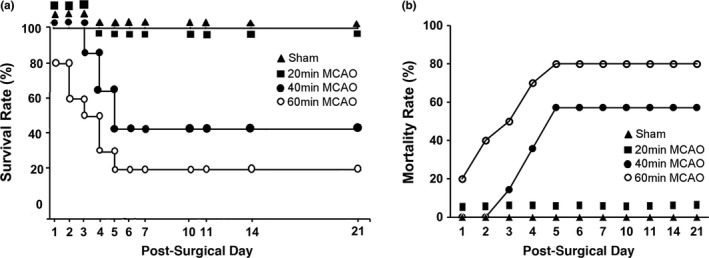
(a) Survival rate. (b) Mortality rate

Brain damage areas were quantitatively analyzed as described in the Methods Section. As shown in Figure 2 below, a few 40‐ and 60‐min MCAO mice were dead for more than 6 hr. As a result, their brain tissue qualities were deteriorated and could not be collected for pathological evaluation. Figure 1b shows the quantitative analysis of brain damage areas of the remaining mouse brains at different planes relative to bregma. The average brain damage areas were : 60 min > 40 min > 20 min MCAO groups in all mouse brain section planes (Fig. 1b). Figure 1c shows the total brain damage volume. Consistently, the 60 min MCAO group had a significantly larger damage volume than the 40 min MCAO group had. In comparison, the 20 min MCAO group had a significantly smaller damage volume than the 40 min MCAO group had.

### 3.2 Survival rates and mortality

Figure 2 shows the survival rate (Figure 2a) and mortality rate (Figure 2b) of the mouse MCAO groups. All sham‐operated control mice (Figure 2a, filled triangle) and 20‐min MCAO mice (Figure 2a, filled square) were survived for all 21 days postsurgically. After 40‐min MCAO, there were about 85.7% mice survived at day 3, 64.3% at day 4, and 42.9% at day 5 postsurgically (Figure 2a, filled circle). All 40‐min MCAO mice alive at 5 days postsurgically survived for the entire 21‐day postsurgical period (Figure 2a, filled circle). After 60‐min MCAO, there were about 80% mice survived at day 1, 60% at day 2, 50% at 3, and 20% at 6 days postsurgically (Figure 2a, open circle). Similar to that of 40‐min MCAO, all 60‐min MCAO mice alive at 6 days postsurgically survived for the entire 21‐day postsurgical period (Figure 2a, open circle). Mortality rate was zero in sham‐operated and 20‐min MCAO group, but was significantly higher in 40‐min MCAO group, and the highest in 60‐min MCAO group (Figure 2b).

### 3.3 Nest‐building activity

**Figure 3:**
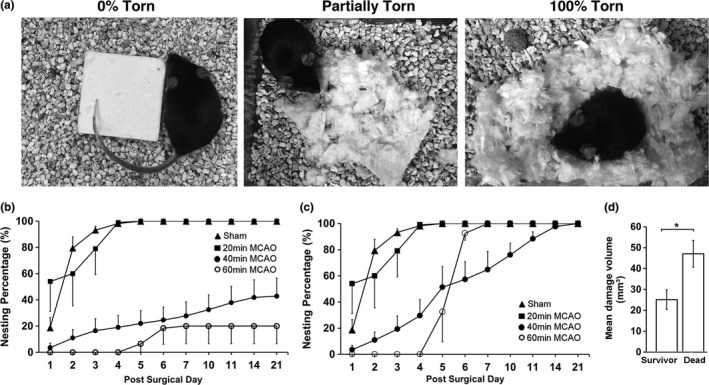
(a) Photographs of the nest‐building levels; (left) 0% turn or completely untorn nestlet, (middle) partially torn nest, and (right) 100% torn nest. (b) Percentage of nest‐building activity calculated with inclusion of mice who died after 40‐ and 60‐min middle cerebral artery occlusion (MCAO) (dead mice = 0%, see Methods). Sham surgery (*n* = 11, filled triangle), 20‐min (*n* = 6, filled square), 40‐min (*n* = 14, filled circle), and 60‐min (*n* = 10, open circle) MCAO groups. (c) Percentage of nest‐building activity calculated with data only from survived mice. (d) The percentage of the damage volume between dead mice (*n* = 8) and mice who survived the entire postsurgical period (*n* = 6) after 40‐min MCAO. Data (b ‐ d) are expressed as mean ± *SEM* for the indicated number of experiments (*n*). Mann–Whitney Wilcoxon analysis was used for the statistical analysis. *p < .05

Nest‐building activity has not been reported as a functional test of stroke deficit. This study developed a methodology for evaluation of nest‐building activity for indexing stroke deficits in the mouse MCAO model. Briefly, the torn and untorn nest materials were weighed. The percentage of torn vs. untorn nest materials was then calculated. Nest‐building activity was quantitatively evaluated daily until the post‐MCAO 21 days. Figure 3a shows a 0% torn or untorn (left), a partially torn (middle), and a 100% torn nestlet, respectively. Figure 3b shows the changes in the nest‐building activity in mice following sham surgery, 20‐min, 40‐min, or 60‐min MCAO, respectively. As shown in Tables 1 and 2, after sham surgery, mice built, in average, 18.73 ± 7.83% of torn nest material or simply nest (hereafter) at day 1, 79.45 ± 8.47% of nest at day 2, and ≥93.18 ± 2.81% of nest from day 3 onward (Figure 3b). Similarly, after 20‐min MCAO, mice built, in average, 54% of nest at day 1, 60% at day 2, 79% of nest at days 3, and ≥99% from day 4 onward. In comparison, after 40 min of MCAO, mice built less than 43% of nest at any given day throughout the entire 21‐day period of reperfusion, significantly lower than those built by mice after either sham surgery or 20 min of MCAO (Figure 3b, Tables 1, 2). Furthermore, after 60‐min MCAO, mice built even lesser nest relative to those after 40‐min MCAO at about 20% level at any given day throughout the 21‐day period of reperfusion (Figure 3b, Tables 1, 2).

**Table 1:** Nest‐building activity: Mean ± *SEM*

Group	Post‐MCAO Day										
	1	2	3	4	5	6	7	10	11	14	21
Sham (*n* = 11)	18.73 ± 7.83	79.45 ± 8.47	93.18 ± 2.81	98.25 ± 0.58	100.0 ± 0.00	100.0 ± 0.00	100.0 ± 0.00	100.0 ± 0.00	100.0 ± 0.00	100.0 ± 0.00	100.0 ± 0.00
20'MCAO (*n* = 6)	54.00 ± 22.7	60.00 ± 24.5	79.00 ± 19.8	99.00 ± 1.00	100.0 ± 0.00	100.0 ± 0.00	100.0 ± 0.00	100.0 ± 0.00	100.0 ± 0.00	100.0 ± 0.00	100.0 ± 0.00
40'MCAO (*n* = 14)	3.57 ± 3.50	10.93 ± 6.37	16.50 ± 9.10	19.00 ± 9.16	22.00 ± 9.94	24.57 ± 9.92	27.79 ± 10.8	32.57 ± 11.2	37.86 ± 12.4	41.86 ± 13.4	42.86 ± 13.7
60'MCAO (*n* = 10)	0.00 ± 0.00	0.00 ± 0.00	0.00 ± 0.00	0.00 ± 0.00	6.50 ± 6.50	18.50 ± 12.4	20.00 ± 13.3	20.00 ± 13.3	20.00 ± 13.3	20.00 ± 13.3	20.00 ± 13.3

MCAO, middle cerebral artery occlusion.

Data are presented as means ±**** *SEM* for the indicated number of experiments (*n*).

**Table 2:** *p* Value of Mann–Whitney Wilcoxon analysis of nest‐building activity

Group	Post‐MCAO Day										
	1	2	3	4	5	6	7	10	11	14	21
Sham vs. 20’	<.060	.283	.317	.226	**‐**	**‐**	**‐**	**‐**	**‐**	**‐**	**‐**
Sham vs. 40’	<.090	**<.001**	**<.001**	**<.005**	**<.002**	**<.004**	**<.004**	**<.004**	**<.006**	**<.020**	**<.030**
Sham vs. 60’	**<.020**	**<.001**	**<.001**	**<.001**	**<.001**	**<.002**	**<.005**	**<.005**	**<.005**	**<.005**	**<.005**
20’ vs. 40’	**<.020**	**<.040**	**<.010**	**<.001**	**<.001**	**<.002**	**<.002**	**<.002**	**<.002**	**<.002**	**<.002**
20’ vs. 60’	**<.010**	**<.010**	**<.010**	**<.001**	**<.001**	**<.001**	**<.003**	**<.003**	**<.003**	**<.003**	**<.003**
40’ vs. 60’	.110	**<.040**	**<.040**	**<.020**	<.088	.181	.200	.200	.181	.143	.126

MCAO, middle cerebral artery occlusion.

KaleidaGraph software (Synergy Software, PA, USA) was used for statistical analysis. “‐” indicates the incomparable nature between the two groups because 100% nest‐building activity in both the sham surgery and 20‐min MCAO group. The bold numbers indicate the statistical signficance (*p*<0.05) between the two groups.

All mice who died after MCAO did not build nest (0%) in any given day before death. Because of that, dead mice after MCAO were considered to build 0% nest (see Methods Section) in the days after their death. Even excluding the data from mice who died after MCAO, there were still significant differences in the nest‐building activities during the post‐MCAO period between the sham surgery group and 40‐min MCAO group (Figure 3c). Furthermore, relative to the infarct volume of mice who died after 40‐ to 60‐min MCAO, the survivors had significantly smaller infarct volume after MCAO (Figure 3d). In other words, the survivors had significantly smaller brain infarct volumes and thus regained some degree of nest‐building activity at the late periods of reperfusion (Figure 3c, open circle).

## 4 DISCUSSION

Because of the poor translation to the clinical application, the Stroke Therapy Academic Industry Roundtable (STAIR) recommends using multiple endpoints for behavioral studies in preclinical stroke research, to demonstrate the clinical relevance. Therefore, development and identification of a group of clinically relevant functional tests may be essential for successfully translating animal studies into the clinical applications. This study characterizes a novel functional test to reflect mouse multifaceted sensorimotor and cognitive coordination ability and well‐being. This is the first report demonstrating that nest‐building activity may be an effective functional test reflecting the long‐term deficit in the mouse model of stroke. We have found that nest‐building deficit directly correlates with mouse stroke severities.

### 4.1 A reproducible mouse stroke model

A reproducible mouse stroke model is essential for the comparison of the mouse functional deficits. In addition to those of keeping consistency in the surgical procedures, and in animal health and experimental conditions, the shape and position of the suture occluder and animal collateral circulation are key factors for the reproducibility of the mouse stroke model. For example, in our hands, when the CCA was temporally ligated, the MCA blood flow was reduced to the 20%‐50% of the preocclusion level in some mice, while 60%‐80% of the preocclusion level in the others, suggesting that the collateral circulation from the contralateral hemisphere varies significantly among different mice (data not shown). There is a strong correlation between silicone‐coating length and infarct size (Guan et al., 2012). In our hands, a silicone‐coating length of 2.0 mm is required to completely occlude the MCA and avoid collateral circulation from the anterior choroidal artery, posterior cerebral artery, and hypothalamic artery.

All mice subjected to the intraluminal procedure had relatively consistent brain damage size in this study. This might be attributable to the very carefully selected smoother silicon‐coated monofilament suture occluder at precisely 0.22‐0.23 mm in diameter and coating tip at 2.0 ± 0.1 mm in length. Another key reason for the consistent MCAO damage might be due to the consistent blood flow to the MCA territory, which was continuously monitored and precisely controlled at below 10% of preocclusion level. This was accomplished by using anesthetized mice during the MCAO period so that the suture occluder position would less likely to move away from the position. Even under carefully controlled anesthetic condition, the MCA blood flow was occasionally jumped to a higher level, suggesting that the suture occluder might indeed be able to move during the MCAO period. Under this situation, the suture occluder position was carefully adjusted to make sure that the MCA blood flow was within less than 10% of the preocclusion level. These measures may be necessary to produce consistent brain damage in this mouse MCAO model.

### 4.2 Nest‐building activity

Mouse natural and spontaneous nest‐building activity is a strong indicator of multifaceted sensorimotor and cognitive function, as well as well‐being, and is sensitive to brain lesions (Jirkof, 2014). The results of the present study support that the nest‐building activity deficit, when including mice who died after MCAO, is likely to become a new and long‐term behavioral phenotype after mouse stroke. This is because that this natural behavioral test can be quantitatively monitored daily and requires minimal effort, while still highly sensitive for indexing the long‐term stroke deficit (Figure 3). Normal mice perform nest‐building activity naturally and autonomously every day without investigator manipulation or enforcement, thus avoiding the variation and bias due to the human handling. The results of the present study further show that deficit in the nest‐building activity correlates well with the stroke severity after mouse MCAO; 20‐min MCAO leads to damage only to a small portion of striatum and thus minimally affected the nest‐building activity, whereas 40‐min and 60‐min MCAO results in corresponding larger brain damage and higher degrees of deficits in the nest‐building activity. Additionally, the nest‐building activity deficit after mouse MCAO is a long‐lasting event. The nest‐building deficit, when including mice who died after MCAO, remains significant at 21 days of recovery after 40‐ and 60‐min MCAO, the longest observation duration in this study.

All mice who died after MCAO did not build nest (0%) in any given day before death. Because of that, dead mice after MCAO were considered to build 0% nest (see Methods Section) in the days after their death. The death is the worst outcome and thus mice who died after MCAO would be the worst performer. This is consistent with that dead mice after stroke have significantly larger infarct volume than mice who survived the same period of MCAO (Figure 3d). In other words, the mice who died after MCAO are those who have significantly severe stroke in the same stroke group. If excluding mice who died after stroke from the 40‐min or 60‐min MCAO group, it will significantly underestimate the nest‐building deficits in these stroke groups. Therefore, it may be reasonable to count the nest‐building activity for mice after their death as the same values as those (0%) before their death.

The quality of the nest (such as the nest size and shape, and nest wall height) may also be characterized. Because of the shape of the nest is often irregular, and height of the different segments of the nest wall may not always be the same, quantitative evaluation of the nest quality may be somewhat subjective.

Another issue may be that some behavioral changes disappear within 3‐5 days after stroke in mouse models because mice adapt the stroke (focal damage) quickly (Freret et al., 2009). For example, the rotarod may not be a test of choice for long‐term evaluation of deficits after mouse MCAO (Freret et al., 2009). In comparison, mice show persistent nest‐building deficit after 40 and 60 min of MCAO (Figure 3b).

Similar to other functional tests, mouse nest‐building activity may be influenced by a number of factors. As shown in Tables 1 and 2, sham‐operated mice perform only about 20% of nest‐building activity in the first day, about 80% in the second day, and about 100% from the fourth day onward after the surgical procedure. This may suggest that the surgical procedure may somewhat affect the nest‐building activity. However, the effect of the surgical procedure and/or anesthesia on the nest‐building activity seems small and short‐lived. Therefore, the surgical procedure and/or anesthesia may not prevent using the nest‐building activity for indexing stroke severity even in the early recovery periods after mouse MCAO (Figure 3). Other environmental factors might also affect mouse nest‐building activity. These may include stress conditions, animal home cage ambient and animal body temperatures, and animal sex, age, and strain, or genetic modifications (Greenberg et al., 2016). For these reasons, to perform the nest‐building behavioral test in stroke animal studies, one may need to keep the surgical procedure as well as environmental conditions as identical as possible to minimize any impacts.

### 4.3 Mechanisms of mouse MCAO‐induced nest‐building deficit

Mouse nest‐building activity involves a complex behavior, requiring coordinating sensorimotor and cognitive activities of pulling, carrying, fraying, push digging, sorting and fluffing of the nest material (Gaskill et al., 2012). Nest‐building activity depends on the striatal and hippocampal function (Szczypka et al., 2001; Fleming et al., 2004; Lin et al., 2007; Sager et al., 2010; Estrada‐Sánchez, Barton, Burroughs, Doyle, & Rebec, 2013). Hippocampus‐ablated rats showed sluggish nest‐building activity (Kim, 1960). These previous studies support the notion that striatal and hippocampal damage after mouse stroke may contribute to the nest‐building deficit. Nest‐building activity is virtually absent in mice deficient in calcium/calmodulin‐dependent protein kinase II (CaMKII) gene (Bachstetter et al., 2014). This may explain the nest‐building deficit after mouse MCAO because CaMKII activity is dramatically reduced in animal models of brain ischemia (Tang, Liu, Kuluz, & Hu, 2004). In addition, a transgenic line of Alzheimer disease displays reduced nest‐building activity (Deacon et al., 2008). Evidence suggests that striatal and hippocampal structural damage, CaMKII depletion and cognitive deficit after mouse MCAO may lead to the nest‐building deficit.

### 4.4 Nest‐building activity as a sensitive and long‐term indicator of mouse stroke functional deficit

To analyze animal functions, one can either measure a natural behavior in an unrestricted manner, or an unnatural behavior in a forced manner. Measuring a natural nest‐building activity in stroked mice may be similar to watching daily activities of stroke patients. The main advantage of quantification of unforced natural behavior is to generate more reliable results potentially reflecting daily activities of stroke patients. Nest‐building activity is natural and spontaneous behavior and has been proposed to represent mouse well‐being (Jirkof, 2014). First, the mouse must be highly motivated and capable of building a nest. This can be witnessed by the fact that a healthy laboratory mouse builds a new nest everyday (Figure 3). Second, nest‐building activity is a multifaceted behavior requiring coordination of sensorimotor and cognitive functions as discussed above. Therefore, to build a nest, a mouse must feel good and have the capability of performing motor, sensory, and cognitive functions. The results of this study show that nest‐building deficiency may be used as a sensitive and long‐term indicator of mouse functional deficit after stroke. Mouse nest‐building activity closely resembles to the “daily living activities” of humans (Lin et al., 2007; Bachstetter et al., 2014). This may be important because a sensitive multifaceted functional and well‐being test that reflects the daily living activities of stroke patients is likely to be a strong clinical indicator of stroke recovery.

In summary, nest‐building activity is performed naturally and spontaneously. It can be easily administered and effortlessly quantified, and can provide reliable results effectively reflecting the measures of the long‐term performance after mouse MCAO. However, it reflects multifaceted functions and animal well‐being, rather than a specific function. Therefore, by combination with pathological data and function‐specific behavioral tests, nest‐building test may be used as a sensitive well‐being indicator for facilitating translation of new therapeutics from bench to bedside.

## CONFLICT OF INTEREST

None declare.
